# Analysis of Cesarean Sections using Robson’s Ten Group Classification System

**DOI:** 10.12669/pjms.37.2.3823

**Published:** 2021

**Authors:** Rashida Parveen, Mehnaz Khakwani, Anum Naz, Rabia Bhatti

**Affiliations:** 1 Rashida Parveen, Department of Obstetrics and Gyne, Unit-II, Nishtar Medical University Hospital, Multan, Pakistan; 2 Mehnaz Khakwani, Department of Obstetrics and Gyne, Unit-II, Nishtar Medical University Hospital, Multan, Pakistan; 3 Anum Naz, Department of Obstetrics and Gyne, Unit-II, Nishtar Medical University Hospital, Multan, Pakistan; 4 Rabia Bhatti, Department of Obstetrics and Gyne, Unit-II, Nishtar Medical University Hospital, Multan, Pakistan

**Keywords:** Robson’s Ten-Group Classification, Cesarean section, Fetal distress

## Abstract

**Objective:**

To analyze trends of CSs and evaluating them according to Robson’s Ten Groups Classification System (TGCS) at a leading government tertiary care hospital of South Punjab, Pakistan.

**Methods:**

This cross-sectional study was conducted at Department of Obstetrics and Gynaecology, Nishtar Medical University Hospital, Multan Pakistan, from October 2019 to March 2020.The study population included a total of 167 women who underwent CS in the hospital during the specified study period. For each case, we collected data regarding maternal characteristics and pregnancy-related information. The dependent variable was Robson classification group.

**Results:**

Overall, mean age was 26.53+5.1 years. Majority of the women, 116 (69.5%) belonged to urban areas of residence, 74 (44.3%) gestational aged between 37-42 years while 108 (64.7%) had history of cesarean section. Most of the patients, 85 (50.9%) turned out to be from TGCS Group-10. Group-5 and Group-1 were the 2nd and 3rd most common group, accounted for 24 (14.4%) and 19 (11.4%) cases respectively. Previous cesarean section (20.4%) and fetal distress (19.8%) were found to be most common indications leading to cesarean section.

**Conclusion:**

As per Robson’s Ten-Group Classification, Group-10 and Group-5 were found to be the most contributing among deliveries done. Previous cesarean section and fetal distress were the most common indications of cesarean section.

## INTRODUCTION

Worldwide, cesarean section (CS) rates have ominously risen in the last few decades.^1^ In cases where spontaneous vaginal delivery (SVD) is not possible or contraindicated, avoiding CS may endanger the lives of mother and the fetus.^2^,^3^ However it is also a reality that CSs are also done without clear indications or with vague indications like obstructed labour, with intact membranes.^4^ CSs are considered to be a life-saving procedures but these are not without risks attached in terms of present or future pregnancies.

Some of the most common short and long term complications associated with CSs are increased chances of maternal morbidity and mortality, increased requirements of blood transfusion, prolonged hospital stays, post-partum infections, retained placenta, stillbirth and post-partum hemorrhage.^5^ This indicates that if not chosen rightly, some women may have needless exposure to these complications while contrary to this, some women might not be getting CS when they are in real need. CS rates are comparatively high among women who are educated (minimum secondary level education), belonging to urban areas of residence or those who have rich socioeconomic status.^6^ In rural areas, unavailability of access to appropriate healthcare facilities and lack of staff and equipment have been found to be leading to increased maternal morbidity and mortality.^7^

In the present scenario, real challenge is to have CS rate low while preserving safety of mother and the newborn intact. For this, constant audits of CSs being performed in healthcare settings are necessary. Three most commonly adopted classifications are “based on primary clinical indications”, “the degree of urgency or absolute need for caesarean delivery”, and “Robson classification”- as frameworks for auditing CS.^8^ Torloni MR et al.^3^ did a systematic review comparing different classifications for CS and concluded that Robson’s10 Groups classification was found to be optimal for monitoring CS. “World Health Organization” has also endorsed Robson’s classification as a “global standard” tool for the monitoring of CS.^9^ The Robson’s classification is known as “Ten Group Classification System (TGCS)”, classifies CSs in ten groups according to different categories of the pregnancy, past obstetrical record, the course of labour and delivery, and the gestational age of the pregnancy (Table-I).

**Table-I:** Robsons Ten Group Classification System.

*Group*	*Description*
1	Group 1: Nullipara, single, cephalic, term pregnancy, spontaneous labour
2	Nullipara, single, cephalic, term, induced labour or planned CS
3	Multipara without uterine scar, single, cephalic, term, spontaneous labour
4	Multipara without uterine scar, single, cephalic, term, induced labour or planned CS
5	Multipara with uterine scar, single, cephalic, term
6	Nullipara, single, Breech presentation
7	Multipara, single, breech, including previous C-Section
8	Multiple Pregnancy
9	Single, abnormal lie, including previous scar
10	Single, Cephalic, Preterm including previous scar

Although, findings of TGCS and its use for analyzing CS rates have been done by researchers in the past,^10^ but there is no study in South Punjab, Pakistan so the exact involvement of various TGCS groups to the overall CS is not knows. Majority of healthcare facilities are available in urban areas of Pakistan and many of the cases undergoing CSs are referred cases but to have an exact audit of CSs according to TGCS is vital to find out the proportion of groups contributing to CSs so this study was planned to trends of CSs and evaluating them according to TGCS at a leading government tertiary care hospital of South Punjab, Pakistan.

## METHODS

This cross-sectional study was conducted at “The Department of Obstetrics and Gynaecology, Nishtar Medical University Hospital, Multan Pakistan”. Approval from “Institutional ethical committee” was taken for this study (Ref. No. 4000, dated 19-02-2020). The study population included a total of 167 women who underwent CS in the hospital during the specified study period. Written consent was taken from all the study participants. Women having laparotomy for uterine rupture or those with missing records were excluded.

For all the women enrolled, maternal history, bio-data, symptomatology, clinical examination, management outcomes, pregnancy-related information (gestational age, fetal presentation, number of fetus and onset of labour) and maternal and fetal outcomes at discharge (complications, APGAR score at five minutes, birth weight) were recorded. The dependent variable was Robson classification group. All the study information was noted on a predesigned proforma.

All completed data was entered in SPSS version 26.0 for analysis. Descriptive statistics of study participants and variables were calculated. The Robson group was assigned based on four obstetric concepts (with their parameters)-category of the pregnancy, previous obstetric history, course of labour and gestational age. Absolute maternal indications included obstructed labour, major antepartum haemorrhage (APH), malpresentation (transverse, oblique and brow) and uterine rupture in hierarchical order. Non-absolute indications included fetal compromise, previous CS, failure to progress, breech, severe pre-eclampsia and eclampsia (with no hierarchy). Results were represented as frequencies, percentages, means and SD.

## RESULTS

During the study interval, a total of 167 deliveries occurred. Overall, mean age was 26.53±5.1 years while most of the women, 152 (91.0%) were between 20 to 35 years of age. Majority of the women, 116 (69.5%) belonged to urban areas of residence, 117 (70.7%) were multigravida, 74 (44.3%) gestational aged between 37-42 years, 108 (64.7%) history of cesarean section and 156 (93.4%) had cephalic fetal presentation (Table-II).

**Table-II:** Characteristics of Study Participants.

*Characteristics*		*Number (%)*
Age (years)	<20	10 (6.0%)
	20-35	152 (91.0%)
	>30	5 (3.0%)
Area of Residence	Urban	116 (69.5%)
	Rural	51 (30.5%)
Gravidity	Primigravida	50 (29.9%)
	Multigravida	117 (70.1%)
Parity	Nulliparous	50 (29.9%)
	Multiparous	117 (70.1%)
Gestational Age (weeks)	<37	93 (55.7%)
	37-42	74 (44.3%)
	>42	0
History of Previous Cesarean Section	None	108 (64.7%)
	1	28 (16.8%)
	>1	31 (18.6%)
Onset of Labour	Spontaneous	83 (49.7%)
	Induction of Labour	4 (2.4%)
	Pre-labour Cesarean Section	80 (47.9%)
Fetal Presentation	Cephalic	156 (93.4%)
	Breech	11 (6.6%)
Apgar Score (at 5 minutes)	≤7	22 (13.2%)
	>7	145 (84.8%)
Birth Weight (grams)	<2500	31 (18.6%)
	2500-4000	128 (76.6%)
	>4000	8 (4.8%)

Distribution of all deliveries performed during the study period according to Robson’s TGCS is shown in Table-III. Most of the patients, 85 (50.9%) turned out to be from Group-10. Group-5 and Group-1 were the 2^nd^ and 3^rd^ most common group, accounted for 24 (14.4%) and 19 (11.4%) cases respectively.

**Table-III:** Distribution of Cesarean Section in terms of Robson’s TGCS.

*Classification*	*Description of Robson’s 10-Groups Classification*	*Number*	*(%) Contribution made by each group to overall CS*
1	Nulliparous, single cephalic, ≥37 weeks, in spontaneous labour.	19	11.4
2	Nulliparous, single cephalic, ≥37 weeks, induced or caesarean section (CS) before labour.	11	6.6
3	Multiparous (excluding previous CS), single cephalic, ≥37 weeks, in spontaneous labour.	11	6.6
4	Multiparous (excluding previous CS), single cephalic, >37 weeks, induced or CS before labour.	4	2.4
5	Previous CS, single cephalic, ≥ 37 weeks.	24	14.4
6	All nulliparous breeches.	4	2.4
7	All multiparous breeches (including previous CS).	5	3.0
8	All multiple pregnancies (including previous CS).	2	1.2
9	All abnormal lies (including previous CS).	2	1.2
10	All single cephalic, <37 weeks (including previous CS)	85	50.9

Indications for cesarean section are listed in Table-IV. Previous cesarean section (20.4%) and fetal distress (19.8%) were found to be most common indications.

**Table-IV:** Indications leading to Cesarean Section in the Present Study (n=167).

*Indications*	*Number (%)*
Previous Cesarean Section	34 (20.4%)
Fetal Distress	33 (19.8%)
Hypertensive Disorders of Pregnancy	10 (6.0%)
Failed Induction of Labour	8 (4.8%)
Cephalopelvic Disproportion	8 (4.8%)
Maternal Requests	7 (4.2%)
Contracted Pelvis	9 (5.4%)
Breech	9 (5.4%)
Abruption	10 (6.0%)
Placenta Previa	9 (5.4%)
Others	30 (18.0%)

## DISCUSSION

World health organization has endorsed CS rate < 15% to balance the risk and benefits of CS. Rising trends in CS rates are feared to implicate lower threshold of labour pains, lesser levels of expertise adopting instrumental deliveries, malpractices, labour induction without indications as well as maternal requests.^11^-^15^ It is very important to keep evaluating CS rates over a period of time and compare it with past data to mark the possible areas of improvement with an aim to lower overall CS rates.^16^,^17^

In the present study, Group-10, Group-5 and Group-1 turned out to be the most prevalent groups accounting for 50.9%, 14.4%, 11.4% cases respectively. Different to our findings, Khan MA et al.^11^ in another local research observed Group-5 and Group-2 to be the most common. Gilani S et al.^18^ found Group-, Group-5 and Group-1 to be the commonest groups showing 30.7%, 17.1% and 21.4% cases respectively. Dhodapkar SB et al.^19^ from India was found to have Group-1, Group-5 and Group-2 as the most prevalent groups accounting for 33.3%, 19.7% and 14.6% cases respectively. All these studies are highlighting the trends according to their own institutional practices regarding handling of delivery cases. There is a need to highlight certain indications leading to specific routes of deliveries which can some what show some similarity especially among the local studies. Khan MA et al.^11^ noted that Group-5, Group-2 and Group-10 were the most contributing group to overall CS rates.^11^ Other researchers from Singapore noted Group-5, Group-2 and Group-10 to be the commonest contributors to CS rates.^20^ One common observation has been that the vaginal delivery is feared to impart uterine rupture among women with previous history of cesarean section. Women undergoing delivery after previous scar have also been thought to opt for CS but this is yet to documented in the local studies. Group 10 followed by Group-5 contributed to majority of the share to total CS cases. Overall, 55.7% of the women belong to gestational age group of below 37 weeks so this can elaborate more than half of the CS cases from Group-10, showing that these women are having complications like hypertensive disorders of pregnancy, decreased fetal movement, fetal distress or intrauterine growth restriction. Preterm labour and preterm rupture of membrane are other commonly found complications among these cases. These results could be representative of the fact that being a leading tertiary care hospital of the region, most cases might be referred to our facility as high risk cases.

### Strength and Limitations of the Study

Present study is the 1^st^ study analyzing trends of CSs and evaluating them according to TGCS a leading government tertiary care hospital of South Punjab, Pakistan. One of the key limitations of this study was that we were unable to record perinatal and maternal outcomes among study participants. As this was a single center study with a comparatively short sample size, results of this study cannot be generalized.

## CONCLUSION

As per Robson’s Ten-Group Classification, Group-10 and Group-5 were found to be the most contributing among deliveries done. Previous cesarean section and fetal distress were the most common indications of cesarean section.

### Authors’ Contribution:

**RP:** Conceived, Data Collection, Responsible for Data’s Integrity and Authenticity.

**MK:** Supervision, Proof Reading.

**AN:** Literature Review, Discussion.

**RB:** Data Collection, Data Analysis.
